# Optimizing Chitin Depolymerization by Lysozyme to Long-Chain Oligosaccharides

**DOI:** 10.3390/md19060320

**Published:** 2021-05-31

**Authors:** Arnaud Masselin, Antoine Rousseau, Stéphanie Pradeau, Laure Fort, Rodolphe Gueret, Laurine Buon, Sylvie Armand, Sylvain Cottaz, Luc Choisnard, Sébastien Fort

**Affiliations:** 1 Univ. Grenoble Alpes, CNRS, CERMAV, 38000 Grenoble, France; arnaud.masselin@airliquide.com (A.M.); antoine.rousseau@cermav.cnrs.fr (A.R.); stephanie.pradeau@cermav.cnrs.fr (S.P.); laurine.buon@cermav.cnrs.fr (L.B.); sylvie.armand@cermav.cnrs.fr (S.A.); sylvain.cottaz@cermav.cnrs.fr (S.C.); 2 Univ. Grenoble Alpes, CNRS, DCM, 38000 Grenoble, France; laure.fort@univ-grenoble-alpes.fr (L.F.); rodolphe.gueret@univ-grenoble-alpes.fr (R.G.); 3 Univ. Grenoble Alpes, CNRS, DPM, 38000 Grenoble, France; luc.choisnard@univ-grenoble-alpes.fr

**Keywords:** chitin oligosaccharides, chemo-enzymatic synthesis, lysozyme, response surface methodology

## Abstract

Chitin oligosaccharides (COs) hold high promise as organic fertilizers in the ongoing agro-ecological transition. Short- and long-chain COs can contribute to the establishment of symbiotic associations between plants and microorganisms, facilitating the uptake of soil nutrients by host plants. Long-chain COs trigger plant innate immunity. A fine investigation of these different signaling pathways requires improving the access to high-purity COs. Here, we used the response surface methodology to optimize the production of COs by enzymatic hydrolysis of water-soluble chitin (WSC) with hen egg-white lysozyme. The influence of WSC concentration, its acetylation degree, and the reaction time course were modelled using a Box–Behnken design. Under optimized conditions, water-soluble COs up to the nonasaccharide were formed in 51% yield and purified to homogeneity. This straightforward approach opens new avenues to determine the complex roles of COs in plants.

## 1. Introduction

Agriculture in the 21st century faces multiple challenges. The production levels have to be increased to satisfy the food needs of a growing world population as well as to meet the feedstock requirements for a booming green economy (biofuel, biomaterials). Meanwhile, chemical inputs must be reduced to limit their huge environmental footprint. Chitin oligosaccharides (COs) hold great promise in the context of developing a more sustainable agriculture [1]. Chitin, a polysaccharide of β-1,4-linked *N*-acetyl-glucosamine units, is an abundant, albeit underexploited, biomass resource on earth. Every year about 100 billion tons [2,3] are produced by crustaceans, mollusks, insects, and fungi however, chitin is discarded in massive amounts (6–8 million tons/year) as waste from the seafood industry [4,5]. Chitin has high recycling potential, not only as a biocompatible and biodegradable material, but also as a source of biologically active oligosaccharides. For instance, COs have been reported to possess anticancer and anti-inflammatory activities [6]. However, the most thoroughly investigated activities of COs are, by far, those contributing to plant defense and plant growth. COs, according to their degree of polymerization present different activities in plants that led to differentiate them into two classes. COs up to the pentasaccharides are commonly referred to as short-chain COs, while higher oligomers are referred to as long-chain COs. Long-chain COs have long been known as plant elicitors. They are recognized as microbe-associated molecular patterns and trigger immunity signaling in several species, with CO-VIII being the most active [7,8,9,10]. Short-chain COs induce oscillations of the calcium concentration in the plant cell nucleus, which is a hall-mark of early arbuscular mycorrhizal and rhizobial symbioses signaling [11,12,13]. Mutual-istic relationships between a host plant and nitrogen-fixing bacteria or arbuscular mycor-rhizal fungi enhance the supply of essential nutrients such as nitrogen, potassium or wa-ter to the host. These symbioses are of high interest in the development of more sustainable agricultural practices. Although only chitintetraose (CO-IV) and chitinpentaose (CO-V) were known to activate symbiosis signaling until very recently, a recent work has established that all COs from CO-IV to CO-VIII have this ability [14]. 

In spite of continued efforts and the recent characterization of a high-affinity receptor for long-chain COs [15], the perception and transduction of COs remain poorly under-stood. Recently, there have been major advances in the discrimination between long-chain COs and lipochitin oligosaccharides in leguminous plants [16]. However, the role of COs in the balance between symbiosis activation and immunity activation in plants remains to be elucidated. Improving the access to long-chain COs up to CO-VIII with high purity is needed to improve the understanding of their biological activities. In addition to basic re-search, the development of green and cost-efficient processes can help meet the needs for organic fertilizers. 

Several research groups have addressed the production of COs either by chemical or by enzymatic methods [17]. The versatility of chemical syntheses is impaired by a huge number of steps [18]; nevertheless, the preparation of a chitosan dodecamer [19] was efficiently achieved in 1993. Since then, no other synthesis of such long oligomers has been reported. A recent report on efficient automated preparation of COs up to CO-VI [20] suggests that access to higher oligomers could also be possible. To date, only acid hydrolysis of colloidal chitin has allowed the preparation by chemical means of COs up to the decasaccharide with a yield of 0.2–0.3% (weight fraction) for the longest oligomers [21]. Enzymatic processes that present the advantage of being softer than chemical ones are likely to allow a better control of the distribution of the products while avoiding their denaturation. However, the crystallinity and insolubility in aqueous media of chitin are barriers to enzyme action. Although much is known about chitinases and related enzymes, efficient conversion of chitin to well-defined oligosaccharides remains a challenge [22]. Syntheses using the transglycosylation activity of wild-type [23] or mutant chitinases [24,25,26] were efficiently achieved; however, the preparative synthesis of long-chain COs was impeded by hydrolysis and disproportionation side activities. The necessity to use substrates issued from chitin depolymerization and the low level of expression of chitinases are additional drawbacks. Nevertheless, efficient transglycosylation of chitinbiose by lysozyme into CO-VI, CO-VII, and higher oligomers was achieved using a buffer containing a high concentration of ammonium sulfate [27].

Among enzyme candidates, hen egg-white lysozyme [28] (HEWL) indeed presents several advantages. HEWL is an industrial enzyme that is commercially available at low cost in large quantities. It is active in a large range of conditions (buffers, temperature), and displays a large substrate promiscuity [29,30,31]. Whereas the natural substrate of HEWL is the bacterial cell wall peptidoglycan, a polymer of *N*-acetyl-glucosaminyl-β-1,4-*N*-acetyl-muramic acid repeating units, HEWL also hydrolyses chitin [29,32] and chitosan [33,34,35], albeit less efficiently. Enzymatic depolymerization of chitinous substrates by HEWL has been deeply investigated in the literature in the past. However, most of the work mainly focused on biochemical and mechanistic studies. Heller et al. reported kinetic studies on HEWL degradation of films and hydrogels made of partially deacetylated chitin [36]. Lysozyme degradation of *N*-acylated chitosan derivatives aimed at biomedical applications was used to assess their biodegradability [37]. A combination of NMR and modelling techniques was used to study the lysozyme specificity for acylated and non-acylated glucosamine units at the six different subsites of the enzyme [34]. Amazingly, the ability of HEWL to hydrolyze partially deacetylated chitin has been barely used to pro-duce oligosaccharides. In 1994, Aiba reported the preparation of COs by this approach [38]. Short-chain COs from CO-II to CO-V were produced, but the pentasaccharide was isolated at a yield of less than 2% and with a purity of only 70%. The average yield of the dimer, trimer and tetramer was 16.3, 10.3 and 18% respectively. This result led the author of the study to conclude that the process was useful for the production of CO-II to CO-IV only. To the best of our knowledge, no other example of CO preparation using this method has been reported in the literature to date. 

Optimizing a reaction or chemical process can be a long and complex task. This is particularly true when using enzymes whose activity is strongly influenced by tempera-ture, pH, and ionic strength of the reaction media. The complexity of the substrate is also a major aspect to consider, and the solubility, viscosity, or structure heterogeneity are im-portant features of polysaccharides. The response surface methodology (RSM), based on the design of experiments (DOE), is an efficient way of optimizing chemical processes [39]. RSM is a multivariate statistical technique used to discover the conditions in which to apply a procedure in order to obtain the best possible response in the experimental region studied. This methodology involves the design of experiments and multiple regression analysis as tools to assess the effects of two or more independent variables on dependent variables. One additional advantage of RSM is the possibility of evaluating the quadratic effects of independent variables on the response. This technique is based on the fit of a quadratic polynomial equation to the experimental data to describe the behavior of a set of data. In this way, a mathematical model, which describes the studied process, is generated. The objective is to optimize simultaneously the levels of the studied variables to attain the best possible performance of the process. Central composite design (CCD) and Box-Behnken designs (BBD) are the two main types of response surface designs. Box-Behnken design [40] always have 3 levels per factor, unlike central composite designs which can have up to 5. Thus, BBD is less expensive to run with the same number of factors. Another advantage is that it does not contain combinations for which all factors are simultaneously at their highest or lowest levels. The number of experiments performed under extreme conditions, for which unsatisfactory results might occur is minimized [41]. 

RSM has gained increasing attention in the depolymerization of polysaccharides, in particular of cellulose [42] and starch [43,44]. A few examples applied to chitin hydrolysis have also been reported. Behera et al. [45] used a Taguchi design analysis to optimize the production of *N*-acetyl-glucosamine from colloidal chitin in *Streptomyces chilikensis*. Pan et al. [46] investigated the production of low-molecular-weight chitosan with papain using the Box-Behnken model. Finally, Liu et al. [47] optimized the preparation of CO-VI using a non-commercial chitinase from *Aeromonas schubertii*. The production of hexasaccharide from colloidal chitin (3.5 g/L) reached 42 mg/L, but the recovery and purification of the product remain a crucial issue. 

**Figure 1:**
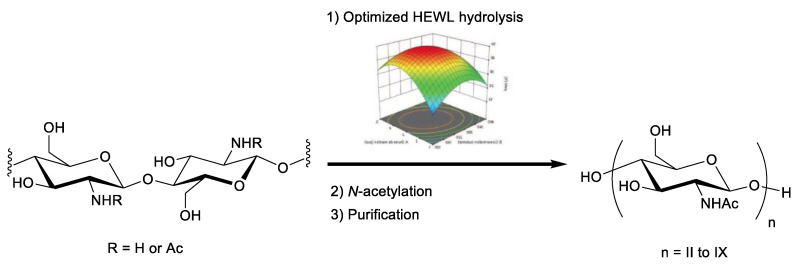
Strategy developed for the production of chitin oligosaccharides (COs) via lysozyme hydrolysis of water-soluble chitin (WSC).

Here, we report the optimized production of short- and long-chain COs by HEWL depolymerization of partially acetylated water-soluble chitin using the response surface methodology (Figure 1). Chitin with an acetylation degree between 0.3 and 0.7 is soluble in aqueous media and therefore more easily processed by enzymes. Our objective was to use such water-soluble chitin (WSC) and optimize HEWL hydrolysis conditions (time, concentration and buffer) to produce both short- and long-chain COs after selective *N*-acetylation. Despite an abundant literature on HEWL, such process has never been report-ed so far.

## 2. Results and Discussion

### 2.1. Initial Screening of HEWL Activity 

RSM allows the optimization of a reaction with a minimum of experiments as long as the key factors are suitably defined. Too many parameters influence HEWL activity; thus, we only considered WSC concentration, degree of acetylation, and the reaction time course for the design of experiments. The choice of appropriate buffer and pH was determined by a preliminary screening of the reaction conditions.

HEWL activity is abundantly documented in the literature; however, optimal buffer and pH differ somewhat according to the substrate and the analytical methods used. With chitin oligosaccharide substrates, the highest hydrolytic activities are observed at a pH between 4.5 and 5.5 [48,49], whereas on *Micrococcus lysodeikticus* cells, the maximum is reached at a pH between 6 and 9 [31,50]. HEWL activity was first assayed by monitoring the fluorescence released from 4-methylumbelliferyl-*N*,*N*′,*N*″-triacetyl-β-chitotrioside (4-Mu-CO-III) in pure water and different buffers (sodium acetate, sodium citrate, potassium phosphate, and (*N*-morpholino)propanesulfonic acid sodium salt (MOPS)). Temperature and enzyme and substrate concentrations as well as ionic strength were kept constant. HEWL activity on 4-Mu-CO-III in water, sodium citrate (pH 5.7), MOPS (pH 6.5), and potassium phosphate (pH 7.7) buffer was relatively low (Supplementary Materials). The highest conversion rates were observed in sodium acetate buffer (pH 5.6) and in potassium phosphate buffer (pH 5.7 and 6.5). This result suggests that in addition to the influence of the pH, the nature of the buffer does have an impact on HEWL activity. As reported in the literature, the highest hydrolysis of 4-Mu-CO-III occurs in acetate and phosphate buffer at pH 5.5 [48]. Surprisingly, HEWL displayed a decreased activity in citrate buffer compared to acetate buffer at the same pH of 5.5. Similarly, HEWL activity at pH 6.5 was lower in MOPS compared to phosphate buffer. It was previously reported that buffer ions have a significant impact on the HEWL solubility in crystallization experiments [51]. The present results suggest that the nature of ions also affects HEWL activity somewhat. Modification of the ionic strength of the buffer did not change HEWL activity substantially (data not shown). Due to significant self-hydrolysis of 4-Mu-CO-III above 40 °C, we did not assess the effect of an increase in temperature.

**Figure 2:**
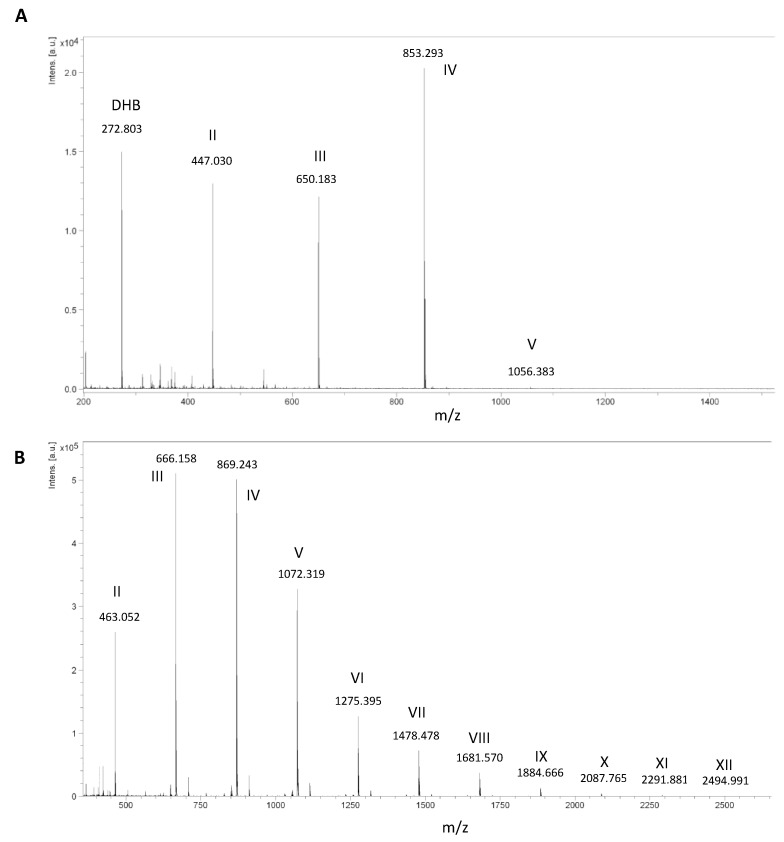
MALDI-TOF spectra of per-*N*-acetylated chitin oligosaccharides (COs) resulting from the hydrolysis of WSC DA 0.42 (10 mg/mL) by HEWL (2.5 mg/mL) in (**A**) sodium acetate (60 mM, pH 5.6) and (**B**) potassium phosphate buffers (60 mM, pH 6.5). Roman numerals represent the degree of polymerization (DP) of COs. Product masses (m/z) correspond to the [M + Na]^+^ species in 2A and [M + K]^+^ in 2B.

To discriminate between acetate and phosphate buffers, we carried out the hydrolysis of WSC at an acetylation degree (DA) of 0.42. The reaction was stopped after 72 h, and the crude oligosaccharide mixture was *N*-acetylated before being analyzed using mass spectrometry (Figure 2A,B). The MALDI-TOF spectrum of the reaction conducted in sodium acetate buffer (pH 5.6) showed the formation of CO-II, CO-III, and CO-IV as the main products and the presence of CO-V in very low amounts. Interestingly, the product distribution was much broader in potassium phosphate buffer (pH 6.5), with the presence of long-chain COs up to CO-XII and an apparent maximum for the tri- and tetrasaccharides. High, but not optimal, HEWL activity seemed to be a good compromise for the production of long-chain COs, hence the phosphate buffer (pH 6.5) was selected for designing the experiments.

### 2.2. Optimization of WSC Hydrolysis

A Box-Behnken design (BBD) was used to optimize the production yield of water-soluble COs. The three selected factors i.e. acetylation degree (DA), substrate concentration (C) and reaction time-course (t), were coded at three levels between −1 and +1, with the values varying as shown in Table 1. 

WSC samples with a DA of 0.32, 0.42 and 0.59 were prepared to ensure both good substrate solubility and significant hydrolysis by HEWL [52]. Although these substrates can be obtained by the chemical deacetylation of chitin, we used a milder and more reproducible method consisting of selective *N*-acetylation of chitosan [53]. Substrate concentrations were defined between 5 and 20 mg/mL to allow full solubilization of the starting material and to provide a sufficient amount of oligosaccharides at the end of the reaction to determine a reliable reaction yield. Finally, the reaction time-course was defined between 24 h and 120 h. Preliminary experiments showed extremely low conversion yields in less than 24 h; however, HEWL retained 90% of its initial activity in phosphate buffer after 4 days (data not shown).

Our experimental design was based on 17 experiments, including 5 replications with the factors at their central values (design center) to evaluate the pure error, and the experiments were carried out in randomized order (Table 2). HEWL concentration (2.5 mg/mL), temperature (37 °C), and stirring speed (180 rpm) of the reaction were kept constant during all experiments. After reaction workup, *N*-acetylation, and a desalting step, the yield (Y) of crude water-soluble COs was determined.

From the data reported in Table 2, the BBD quadratic model coefficients were estimated in accordance with the established multilinear regression (MLR) procedure, which allows fitting the observed response to the analytical model [54]. The quadratic model including all coefficients was refined using the stepwise technique [55]. This procedure involves removing each eligible coefficient one by one to find the model that best fits the data according to the lowest Akaike information criterion (AICc) likelihood statistics criteria [56,57]. From the observed data, the best reduced model (Equations (1) and (2)) for the experiment in terms of the natural values was obtained with an AICc value of 122.98:

(1) $$Y=49.08+3.5t-2C+1.25DA-4.75tC-14.67t^{2}-11.68C^{2}-17.15DA^{2}$$

(2) $$R^{2}=0.9421\;R_{Adjusted}^{2}=0.8971\;R_{Predicted}^{2}=0.7199$$

An analysis of variance (ANOVA) was carried out to determine the statistical significance of the fitted quadratic model and the coefficient terms (Table 3).

**Figure 3:**
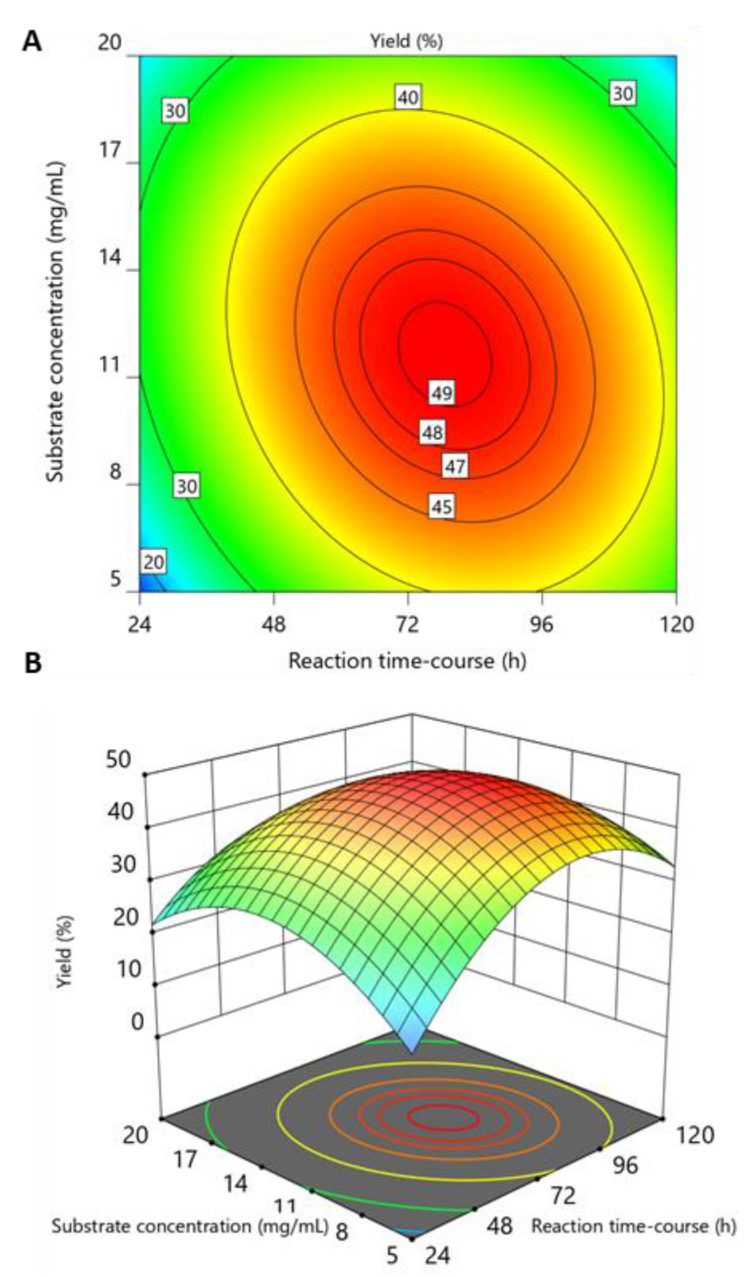
(**A**) Contour plot and (**B**) Response surface plot. The acetylation degree was set to 0.42 (center level) in both plots. The response of the target variable (yield) is color-coded, ranging from blue (low yield) to red (high yield).

Considering the ANOVA table output, if the calculated *F* value (*F*) is larger than *F* critical value founded from the *F* Distribution, the null hypothesis can be rejected (H0: model term = 0). However, the Design-Expert software does not report the *F* critical value but the probability *p*-value. The *p*-value determined from the F statistic is the probability your results could have happened by chance. A *p*-value less than 0.05 or *F* > *F* critical value indicates that the selected model term is statistically significant (*t*^2^, *C*^2^, *DA*^2^), and a value greater than 0.05 indicates that the model term did not significantly affect the output of the responses (*t*, *C*, *D*, *DA*, *tC*). As reported in Table 3, the selected reduced model was highly significant, with *p*-value of < 0.0001. Some not significant terms were maintained in the model to respect to the “hierarchy principle” of the model. “Hierarchy” in models means that if interactions or quadratic terms are present in the model, it is necessary to include all the lower-level effects involved in the interaction or quadratic terms. The model fit statistics (*R*^2^ = 0.9421, *R*^2^_Adjusted_ = 0.8971 and *R*^2^_Predicted_ = 0.7199) indicated that the refined model had high regression accuracy. The model *Adequation Precision* value (11.2) was greater than 4, indicating an adequate signal-to-noise ratio. Considering the quality of these coefficients, we decided to not consider the lack of fit as detected in the ANOVA (*p*-value = 0.0063, Table 3). Finally, the model could be used to predict responses within the given range of factors. As an illustration, a contour plot and a response surface plot for this model, in terms of the actual variables, are shown in Figure 3.

### 2.3. Validation of the Model and Optimized Synthesis of CO-II to CO-IX

**Figure 4:**
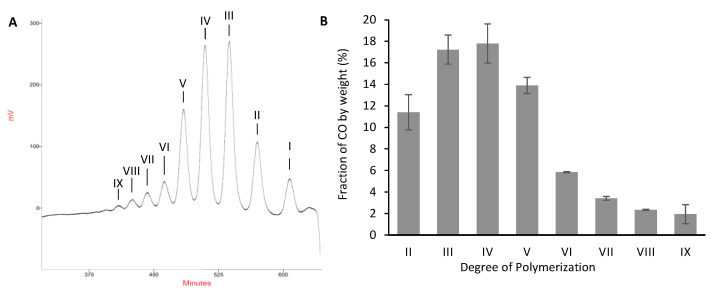
(**A**) Chromatogram of an optimized chitin oligosaccharides (COs) mixture purified using size-exclusion chromatography on Superdex S30 columns; (**B**) Weight fraction of isolated CO (purification was performed in triplicate).

In addition to the experimental design points in Table 2, an additional trial with a single combination of factor settings was added to simultaneously check the accuracy of the model under new experimental conditions and to find the maximum yield. A Nelder-Mead simplex-algorithm-based numerical optimization was used to identify the best sub-set of variable setting combinations that maximize the desirability function [58,59]. The reaction conditions determined using the algorithm predicted a yield of 48% after 79 h at a substrate concentration of 11.2 mg/mL with chitin DA 0.47. Preparative synthesis of COs was carried out in triplicate on a gram scale under the optimal conditions predicted by the model. A crude mixture of water-soluble COs with the expected molecular weight distribution and an average yield of 51 ± 5% was obtained. Although the reaction had been scaled up, the theoretical and experimental yields were very close (within a 95% predic-tion interval), supporting the predictive ability of the model. Purification using size-exclusion chromatography allowed the isolation of each oligosaccharide up to the nonasaccharide (Figure 4A) with excellent purity as attested by mass spectra and chromato-graphic analyses performed on the purified molecules (Supplementary Materials). As a whole, the 75% recovery rate of CO-II to CO-IX after purification (Figure 4B) was very high. Some non-specific adsorption of the highest DP COs onto the filter and onto the chromatographic support, as well as a small fraction of non-isolated GlcNAc, accounted for the unavoidable loss of material. Noteworthily, in addition to the excellent repeatability of the reaction, purification was also highly reproducible as exemplified by the low standard deviation values (Figure 4B). Depolymerization of partially deacetylated chitin by HEWL was previously shown to allow the preparation of short-chains COs (CO-II to CO-IV) with an average yield of about 45% [38]. In the present work we demonstrate that a BBD optimization process also allows the production of long-chain COs while increasing the yield to 51%. For comparison, non-purified mixtures of long-chain COs were obtained with a yield of 32% by transglycosylation reactions using HEWL and CO-III as substrate [60]. Considering the cost of CO-III compared to partially deacetylated chitin, the present process offers an economical and efficient access to both short-chain and long-chains COs.

## 3. Materials and Methods

### 3.1. Material and Equipment

Commercially available low-molecular-weight chitosan (Ref 448869), HEW Lysozyme (Ref 62970, ~100,000 U/mg, 1 U corresponds to the amount of enzyme which decreases the absorbance at 450 nm by 0.001 per minute at pH 7.0 and 25 °C (*Micrococcus luteus*, ATCC 4698, as substrate), and 4-Methylumbelliferyl-*N*,*N*′,*N*″-triacetyl-β-chitotrioside (4-Mu-CO-III) were purchased from Sigma-Aldrich ((Sigma-Aldrich, Saint-Quentin-Fallavier, France). Preparative size-exclusion chromatography was performed with three Hiload Superdex 30 columns (10–300GL) (GE Healthcare, Uppsala, Sweden) in series using refractive index detection. COs mixtures (10 mg/mL) in ultrapure water were filtered through a 0.2 µm membrane before injection (10 mL). Elution was performed with 0.1M carbonate ammonium at a flow rate of 1.2 mL/min. NMR spectra were recorded in D_2_O with a Brucker Avance III 400MHz spectrometer (Bruker, Wissembourg, France). The solvent residual peak of D_2_O at 4.25 ppm (353 K) was used as internal standard. Chemical shifts δ are reported in ppm relative to the solvent residual peak. MALDI-TOF spectra were recorded with a Brucker Autoflex Speed (Bruker, Wissembourg, France) using a 2,5-dihydroxybenzoic acid (DHB) matrix. Fluorescence spectra were recorded at 20 °C with a CLARIOstar microplate reader (BMG Labtech SARL, Champigny s/Marne, France). 

### 3.2. Determination of HEWL Activity with 4-Mu-CO-III

In a plastic microtube (1.5 mL)**,** a solution of 4-Mu-CO-III (27 µM) and HEW lysozyme (0.13 mg/mL) in buffer solution (400 µL) was agitated for 5 h at 40 °C in an Eppendorf ThermoMixer (Eppendorf France, Montesson, France). Every hour, an aliquot (50 µL) was withdrawn, and the reaction was stopped by the addition of glycine-sodium hydroxide buffer (0.1 M, pH 10, 150 µL). The fluorescence caused by the release of 4-methylumbelliferone at an excitation wavelength of 360 nm and emission wavelength of 450 nm was determined against a blank solution of 4-Mu-CO-III incubated in the same conditions but without lysozyme.

### 3.3. Box-Behnken Design

The response surface methodology (RSM) was employed to maximize the formation of water-soluble COs by depolymerization of WSC with lysozyme, followed by selective *N*-acetylation. A Box–Behnken design (BBD) was applied to study the effects of three process variables, namely, reaction time course (t), concentration of water-soluble chitin (C), and degree of acetylation (DA) of WSC, as specified in Table 1.

A total of 17 experimental sets, which included 12 factorial points and 5 centering points, were adopted. The three experimental variables were designed at three levels coded with a plus sign (+1; high value), zero (0; central value), or a minus sign (−1; low value). The coded values of these factors were obtained using Equation (3):

(3) $$x_{i}=\frac{X_{i}-X_{0}}{∆X_{i}}$$

where *x_i_*, *X_i_*, and *X_0_* (*i* = 1–3) represent the coded, real, and central values of the independent variable, respectively, and Δ*Xi* = (variable at high level − variable at low level)/2, denotes the step change value.

A model equation based on the quadratic polynomial given by RSM was used to reveal interactive effects between experimental variables, to optimize the reaction process, and to predict the yield of COs. The model equation may be expressed as:

(4) $$Y=\beta_{0}+\sum^{\;}\beta_{ii}x_{i}^{2}+\sum^{\;}\beta_{ij}x_{i}x_{j}$$

where *Y* is the predicted response (i.e., product yield), *x_i_* and *x_j_* are the coded levels of the independent variables, and *β_0_*, *β_i_*, *β_ii_*, and *β_ij_* denote the regression coefficients representing the offset, linear, quadratic, and interaction terms, respectively. The Design-Expert software version 12.0.3.0 (State Ease, Inc., Minneapolis, MN, USA) was used to analyze the experimental data, perform the analysis of variance (ANOVA), and evaluate the regression equation. Accordingly, the fitted polynomial equation may further be expressed in terms of response surface and contour plots to facilitate visualization of the correlations between the response and the experimental variables at various coded levels and to infer optimized process conditions. The coefficient of determination (*R*^2^) may be used to evaluate the accuracy and applicability of the second-order multiple regression model. The significance of its regression coefficient was checked with the *p*-value.

### 3.4. Preparation of Water-Soluble Chitin (WSC) DA 0.32/0.42/0.47/0.59

Commercially available chitosan DA 0.2 (1 g) was dissolved in a solution of 1% (*v*/*v*) AcOH in water (75 mL) and diluted with MeOH (75 mL). Acetic anhydride (110 μL/0.2 equiv. per amino function, 200 μL/0.35 equivalents, 260 μL/0.47 equivalents or 400 μL/0.7 equivalents, depending on the target DA (0.32/0.42/0.47/0.59 respectively), was added whilst the solution was vigorously stirred. After 24 h at room temperature, the solution was concentrated, co-evaporated twice with toluene, and freeze-dried.

For NMR analysis, 10 mg of WSC was dissolved in a D_2_O/TFA solution (1/0.01 *v*/*v*, 1 mL). The solution was evaporated to dryness, and the residue was dissolved in D_2_O.

*DA* was determined by ^1^H NMR with Equation (5):

(5) $$DA=\frac{H-1^{GlcNAc}}{H-1^{GlcNAc}+H-1^{GlcN}\;}$$

where *H*-1*^GlcN^* and *H*-1*^GlcNAc^* correspond to the integration of the glucosamine and *N*-acetyl-glucosamine anomeric signals, and *H*-1*^GlcNAc^* is arbitrarily normalized to 1.

As an illustration, NMR characterization of WSC DA 0.42 is reported below, while those of others degree of acetylation are provided in Supplementary Materials.

WSC DA 0.42: ^1^H NMR (400 MHz, D_2_O, 353 K) *δ* 5.01 (m, 1.34H, *H*-1*^GlcN^*), 4.73 (m, 1H, *H*-1*^GlcNAc^*), 4.03–3.72 (m, 13.1H, *H*-2,3,4,5,6*^GlcNAc^*, *H*-3,4,5,6*^GlcN^*), 3.32 (m, 1.39H, *H*-2*^GlcN^*), 2.21–2.16 (s, 3.08H, COCH_3_).

### 3.5. Experimental Design of WSC Hydrolysis by HEWL

WSC of each DA (100, 250, or 400 mg) was placed in a glass cylindrical conical flask (60 mL). Potassium phosphate buffer (60 mM, pH 6.5, 20 mL) was added, and the mixture was agitated for 4 h at 37 °C on an orbital shaking incubator (180 rpm) to fully solubilize WSC. HEW lysozyme (50 mg) was added, and the solution was agitated at 37 °C (24, 72, or 120 h). The reaction mixture was immersed in boiling water and vigorously agitated with a magnetic stirring bar (1500 rpm) for 20 min to inactivate the enzyme. The solution was transferred into a conical centrifuge tube (50 mL) and centrifuged (7000 rpm, 20 °C, 10 min), and the supernatant was transferred into a round-bottom flask (100 mL) and freeze-dried. The solid was dissolved in water (8 mL), then methanol (40 mL) and acetic anhydride (2 mL) were added. The reaction mixture was stirred at room temperature for 24 h before being concentrated and co-evaporated twice with toluene. The residue was dissolved in water (35 mL), transferred into a conical centrifuge tube (50 mL), and centrifuged (7000 rpm, 20 °C, 10 min). After washing the pellet with water (10 mL), the supernatants were transferred into a beaker (50 mL) and desalted by the addition of Amberlite IRA400 (OH^−^) resin (5 g) followed by Amberlite IR120 (H^+^) resin (~5 g) until a neutral pH was obtained. The mixture was agitated on an orbital shaker for 1 h until the conductivity was lower than 20 µS/cm, supplementary resin could be added if necessary. When the conductivity was lower than 20 µS/cm, the solution was filtered and freeze-dried to obtain water-soluble COs.

The yield was then calculated as follow:

(6) $$Y_{\mathrm{COs}}\left(\%\right)=\frac{m_{\mathrm{COs}}\times \left(M_{\mathrm{anhydro}-\mathrm{GlcNAc}}{\times \%}_{\mathrm{GlcNAc}}+M_{\mathrm{anhydro}-\mathrm{GlcN}}\times {\%}_{\mathrm{GlcN}}\;\right)}{m_{\mathrm{WSC}}{\times M}_{\mathrm{anhydro}-\mathrm{GlcNAc}}}\times 100$$

$Y_{\mathrm{COs}}\left(\%\right)$: COs yield, $m_{\mathrm{COs}}$: final mass of soluble COs (mg), $m_{\mathrm{WSC}}$: starting mass of water-soluble chitin (mg), $M_{\mathrm{anhydro}-\mathrm{GlcNAc}}$: 203 g/mol, $M_{\mathrm{anhydro}-\mathrm{GlcN}}$: 161 g/mol.

### 3.6. Optimized Preparation of COs from WSC DA 0.47

WSC DA 0.47 (1 g) was placed in glass cylindrical conical flasks (125 mL). Potassium phosphate buffer (60 mM, pH 6.5, 90 mL) was added to the flasks, and the mixture was agitated for 4 h at 37 °C on an orbital shaking incubator (180 rpm) to fully solubilize WSC. HEW lysozyme (225 mg) was added, and the solution was agitated at 37 °C during 79 h. The reaction mixture was immersed in boiling water and vigorously agitated with a magnetic stirring bar (1500 rpm) for 20 min to inactivate the enzyme. The solution was transferred into conical centrifuge tubes (50 mL) and centrifuged (7000 rpm, 20 °C, 10 min), and the supernatant was transferred into a round-bottom flask (500 mL) and freeze-dried. The solid was dissolved in water (32 mL), then methanol (160 mL) and acetic anhydride (8 mL) were added. The reaction mixture was stirred at room temperature for 24 h before being concentrated and co-evaporated twice with toluene. The residue was dissolved in water (165 mL), transferred into two conical centrifuge tubes (50 mL), and centrifuged (7000 rpm, 20 °C, 10 min). After washing the pellets with water (20 mL), the supernatants were transferred into a beaker (500 mL) and desalted by the addition of Amberlite IRA400 (OH^−^) resin (10 g) followed by Amberlite IR120 (H^+^) resin (~10 g) until a neutral pH was obtained. The mixture was stirred on an orbital shaker for 1 h until the conductivity was lower than 20 µS/cm, supplementary resin could be added if necessary. When the conductivity was lower than 20 µS/cm, the solution was filtered and freeze-dried to obtain water-soluble COs. The average amount of CO obtained for triplicate experiments was 570 mg (yield: 51 ± 5%).

The COs mixture (100 mg) was characterized by mass spectrometry and purified (triplicate) by size-exclusion chromatography, affording CO-II (11.4 ± 1.6 mg), CO-III (17.2 ± 1.3 mg), CO-IV (17.8 ± 1.8 mg), CO-V (13.9 ± 0.7 mg), CO-VI (5.8 ± 0.1 mg), CO-VII (3.4 ± 0.2 mg), CO-VIII (2.3 ± 0.1 mg), CO-IX (1.9 ± 0.8 mg).

## 4. Conclusions

In conclusion, the use of BBD allowed the optimization of partially deacetylated chitin hydrolysis using HEWL. After *N*-acetylation, water-soluble COs were produced in an average yield of 51% in gram-scale reactions. Oligosaccharides up to CO-IX were isolated with high purity with size-exclusion chromatography. The present methodology provides straightforward access to short- and long-chain water-soluble COs, including CO-VIII, an efficient activator of plant innate immunity [16]. These promising results suggest applying the same approach to optimize the production of COs with a specific degree of polymerization.

## Figures and Tables

**Table 1:** List of symbols and coded levels for the corresponding experimental variables and ranges adopted for the BBD design.

Independent Variables	Symbols	Units	Code Levels		
			−1	0	+1
Acetylation degree	DA	%	0.32	0.42	0.59
Substrate concentration	C	mg/mL	5	122.5	20
Reaction time-course	t	h	24	72	120

**Table 2:** Box-Behnken design matrix for the three factors (DA, C, t; see Table 1) with three levels each and including five replications with all three factors at their central values.

Experiment	DA	C	t	Response Y (Yield)
1	0		−1	16%
2	0	−1	+1	34%
3	0	+1	−1	18%
4	0	+1	+1	17%
5	−1	0	−1	15%
6	−1	0	+1	14%
7	+1	0	−1	14%
8	+1	0	+1	26%
9	−1	−1	0	16%
10	−1	+1	0	25%
11	+1	−1	0	25%
12	+1	+1	0	15%
13	0	0	0	47%
14	0	0	0	49%
15	0	0	0	47%
16	0	0	0	46%
17	0	0	0	49%

**Table 3:** Analysis of variance for the quadratic model (partial sum of squares—Type III).

Source	Sum of Squares	DF	Mean Square	*F*	*p*-Value
Model	3037.77	7	433.97	20.92	<0.0001
t	98.00	1	98.00	4.72	0.0578
C	32.00	1	32.00	1.54	0.2456
DA	12.50	1	12.50	0.6026	0.4575
tC	90.25	1	90.25	4.35	0.0666
t^2^	906.76	1	906.76	43.71	<0.0001
C^2^	573.92	1	573.92	27.67	0.005
DA^2^	1040.94	1	1040.94	50.18	<0.0001
Residual	186.70	9	20.74	/	/
Lack of fit	179.50	5	35.90	19.94	0.0063
Pure error	7.20	4	1.80	/	/
Cor total	3224.47	16	/	/	/

## Data Availability

Data is contained within the article or Supporting Information.

## Supplementary Materials

The following are available online at https://www.mdpi.com/article/10.3390/md19060320/s1. Determination of HEWL activity in various reaction media using a fluorescence assay. ^1^H NMR spectra of chitin DA 0.32/0.42/0.47/0.57. MS spectra and HP-SEC chromatograms of pure CO-VII, CO-VIII, and CO-IX.
