# Supplementary material for: Optimizing Chitin Depolymerization by Lysozyme to Long-Chain Oligosaccharides
Source: Mar Drugs. 2021 May 31;19(6):320. doi: 10.3390/md19060320 (PMC8229320; doi:10.3390/md19060320)
Supplement: Supplementary file 1 [file marinedrugs-19-00320-s001.zip › marinedrugs-1245577-supplementary.pdf]

Supporting Information

Optimizing chitin depolymerization by lysozyme to long-chain oligosaccharides

Arnaud Masselin,[1] Antoine Rousseau,[1] Stéphanie Pradeau,[1] Laure Fort,[2] Rodolphe Gueret,[2] Laurine Buon,[1] Sylvie Armand,[1] Sylvain Cottaz,[1] Luc Choisnard[3] & Sébastien Fort[1]

[1] Univ. Grenoble Alpes, CNRS, CERMAV, 38000 Grenoble, France

[2] Univ. Grenoble Alpes, CNRS, DCM, 38000 Grenoble, France

[3] Univ. Grenoble Alpes, CNRS, DPM, 38000 Grenoble, France

Chromatography

Analytical size exclusion chromatography was performed with two Superdex peptide 10/300GL columns in series using refractive index detection. COs mixtures (10 mg/mL) in ultrapure water were filtered on a 0.2 µm membrane before injection (10 mL). Elution was performed with 0.1 M NaCl at a flow rate of 0.4 mL/min.

Nuclear Magnetic Resonance (NMR)

NMR spectra were recorded in D_2O with a Brucker Avance III 400MHz spectrometer. The solvent residual peak of D_2O at 4.25 ppm (353 K) was used as internal standard. Chemical shifts δ are reported in ppm relative to the solvent residual peak.

Mass spectrometry

MALDI-TOF spectrum were recorded with a Brucker Autoflex Speed using 2,5-dihydroxybenzoic acid (DHB) matrix.

Determination of HEWL activity

Fluorescence spectra were recorded at 20 °C with a CLARIOstar microplate reader equipped with thermostated cell holder.

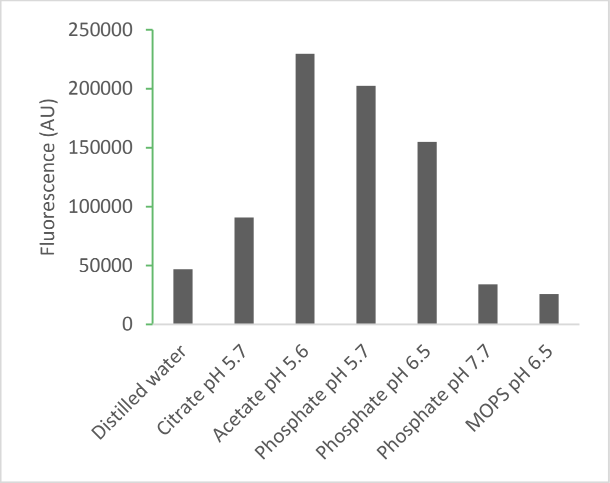

Fluorescence assay of hen egg-white lysozyme (HEWL) (0.13 mg/mL) activity on 4-Mu-CO-III (27 µM) after 5 h at 37°C in distilled water or various buffers (60 mM)

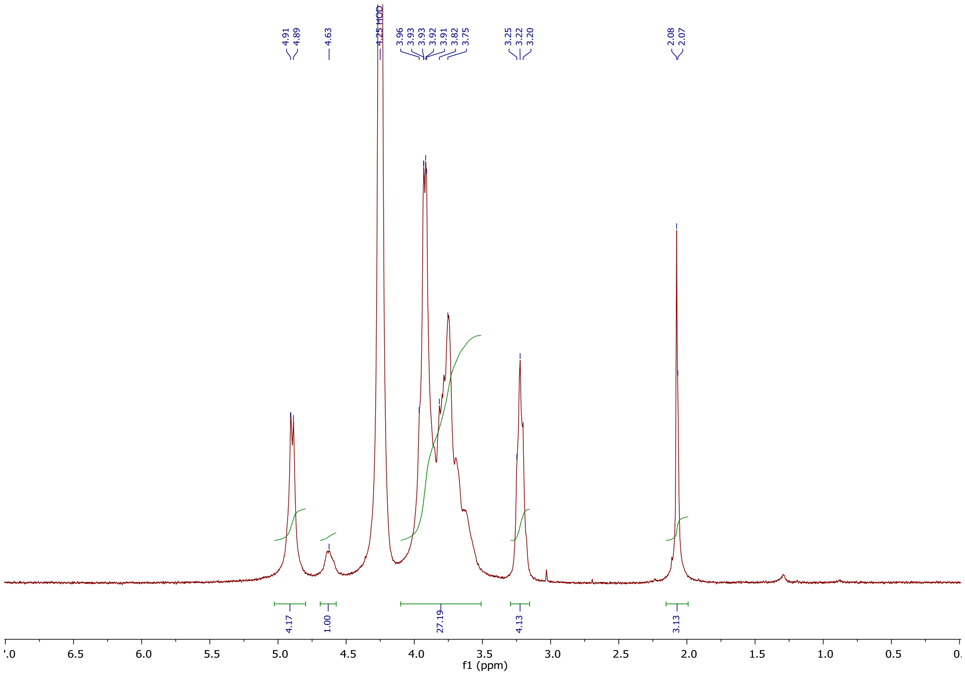

^{1}H NMR Chitosan (Sigma, DA 0.2) (D_2O, 353 K)

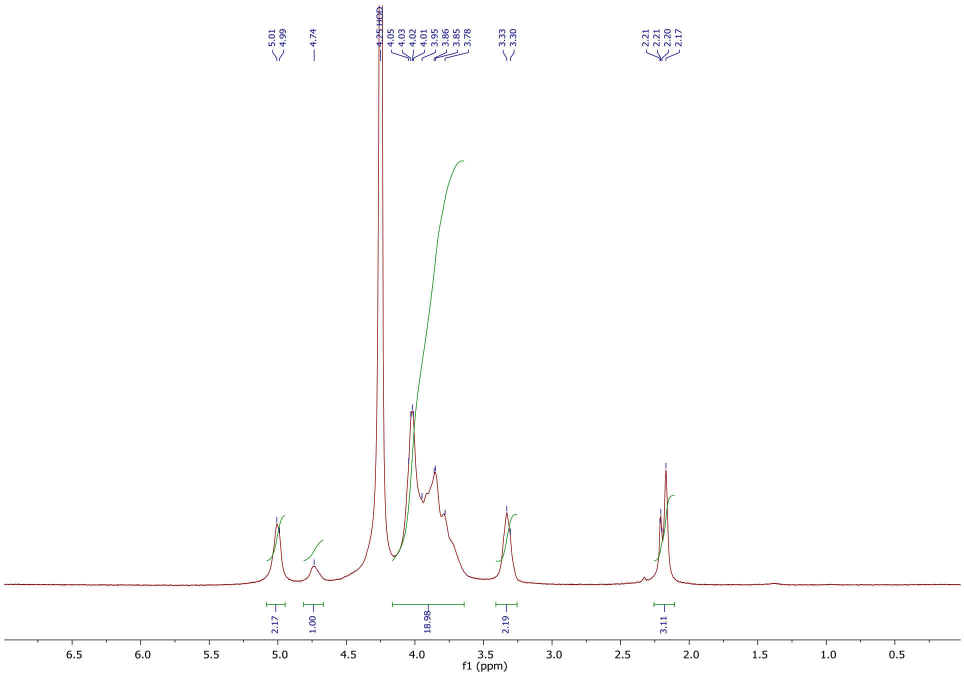

^{1}H NMR Chitin DA 0.32 (D_2O, 353 K)

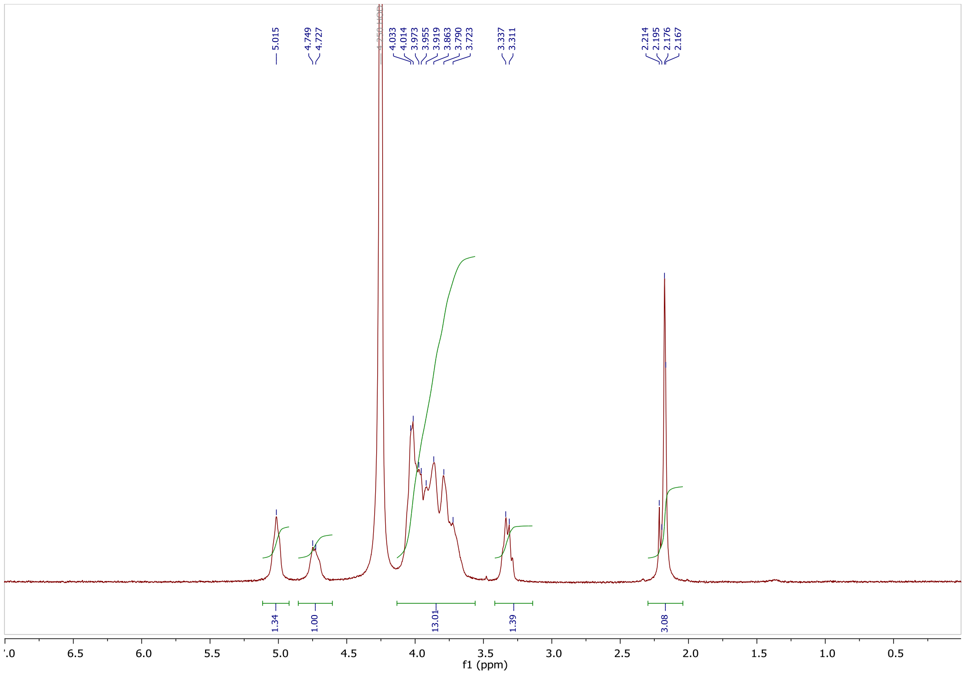

^{1}H NMR Chitin DA 0.42 (D_2O, 353 K)

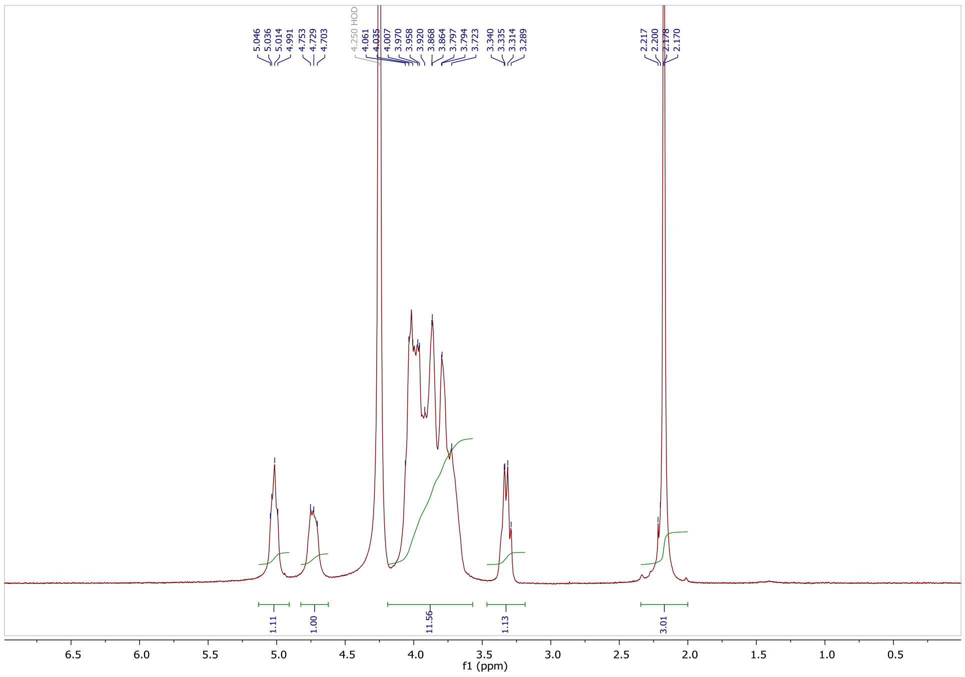

^{1}H NMR Chitin DA 0.47 (D_2O, 353 K)

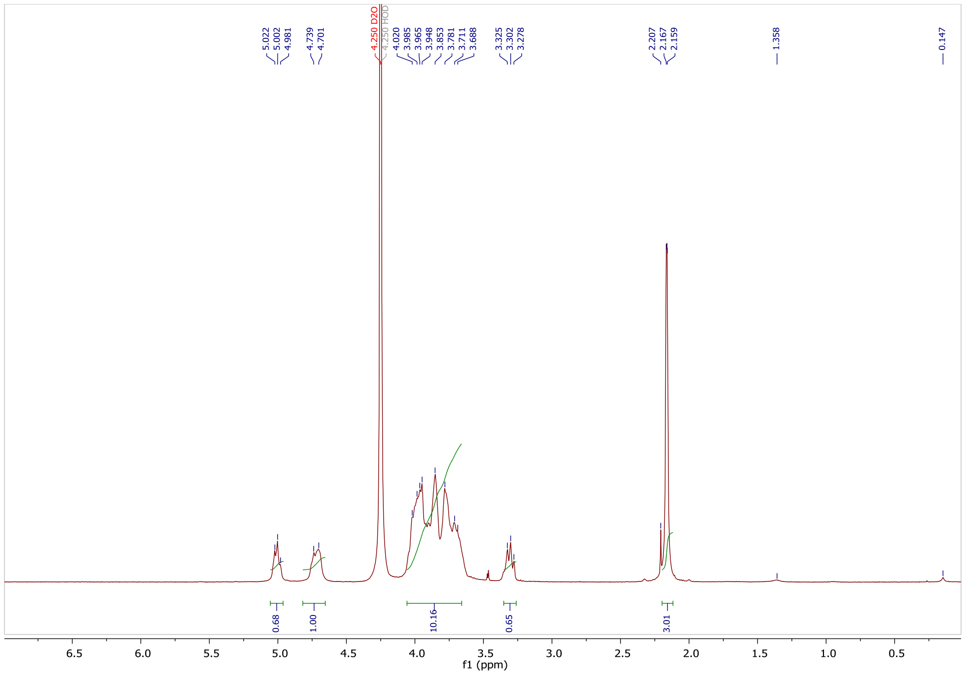

^{1}H NMR Chitin DA 0.59 (D_2O, 353 K)

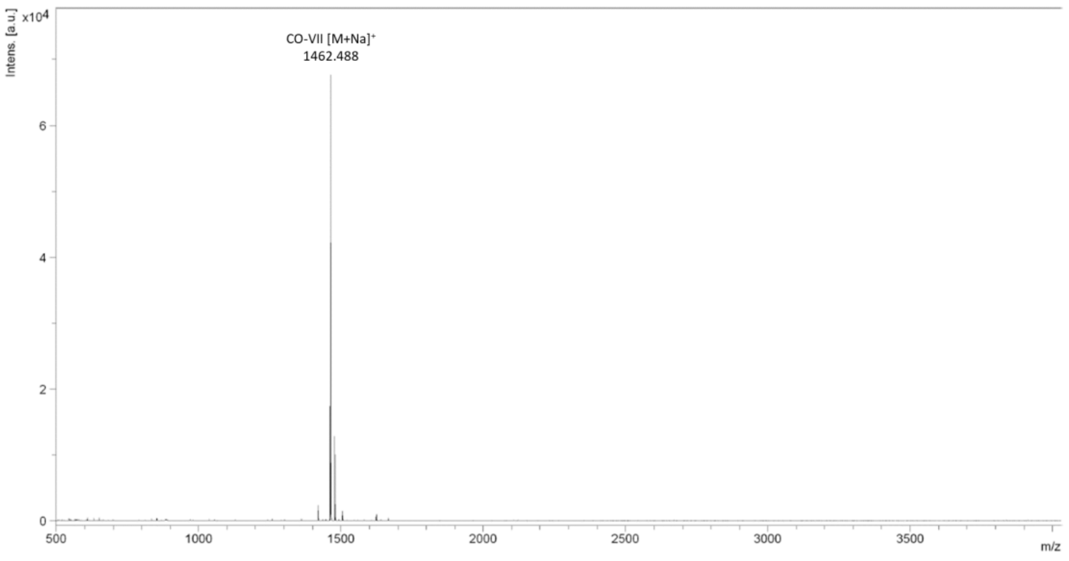

MALDI-TOF mass spectrum of purified CO-VII (positive mode, DHB matrix)

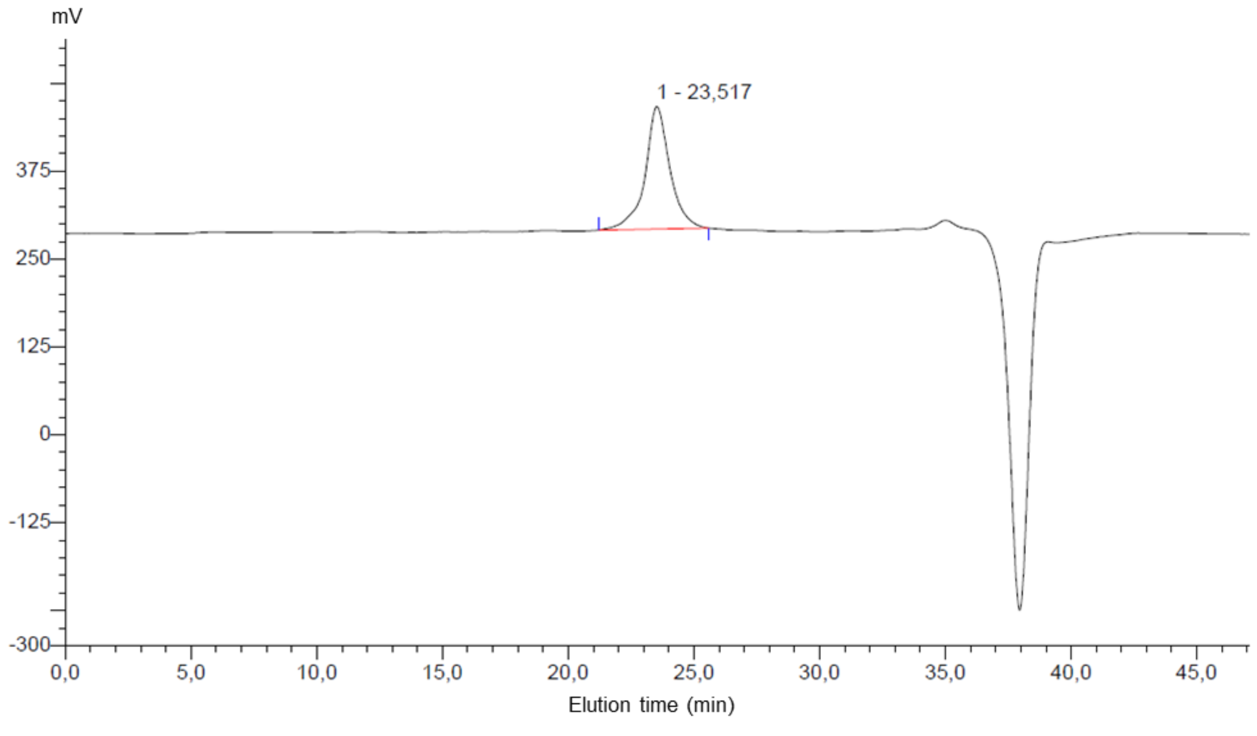

Size-exclusion chromatography profile of purified CO-VII

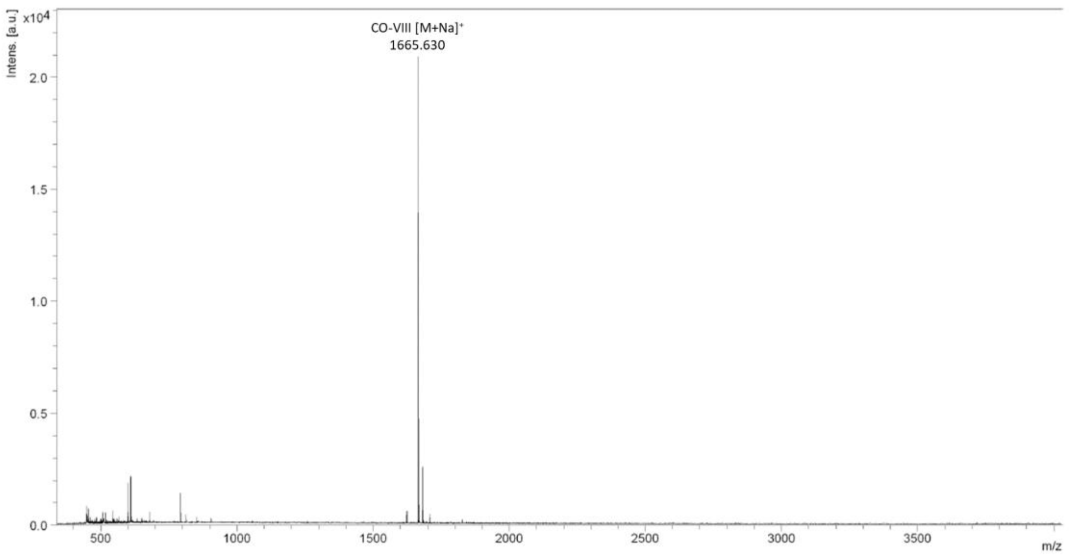

MALDI-TOF mass spectrum of purified CO-VIII (positive mode, DHB matrix)

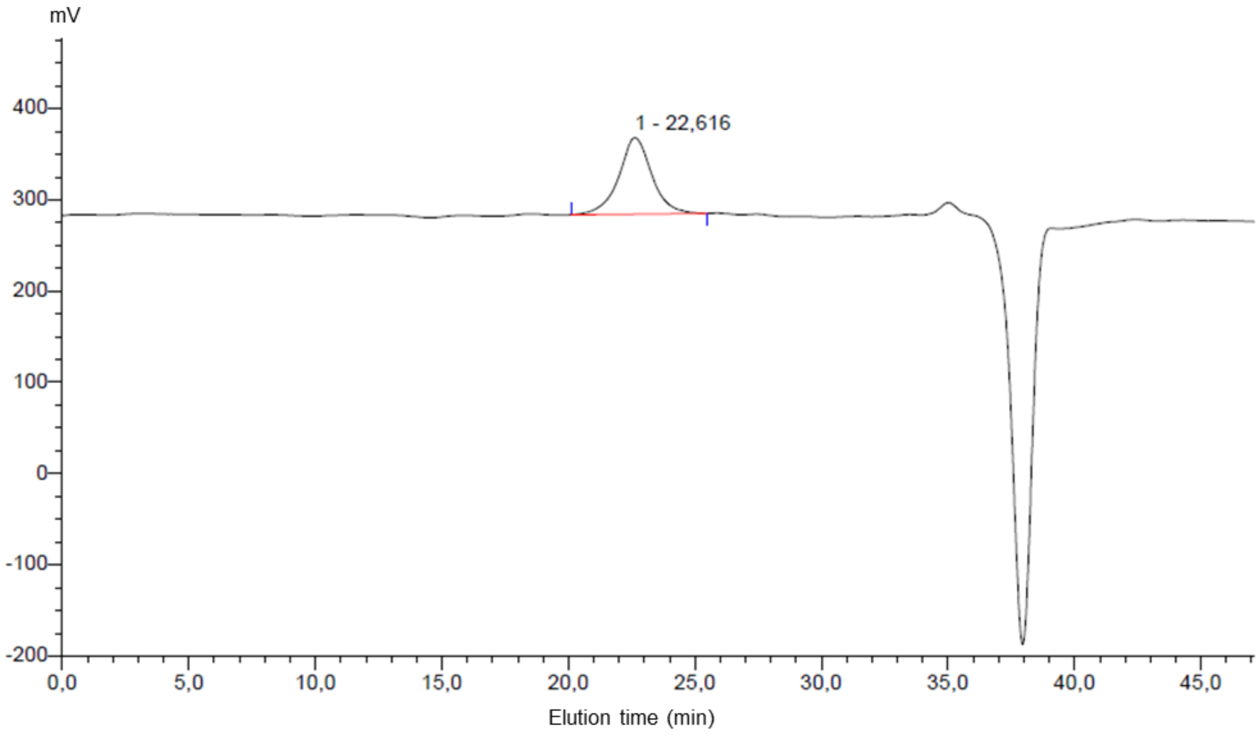

Size-exclusion chromatography profile of purified CO-VIII

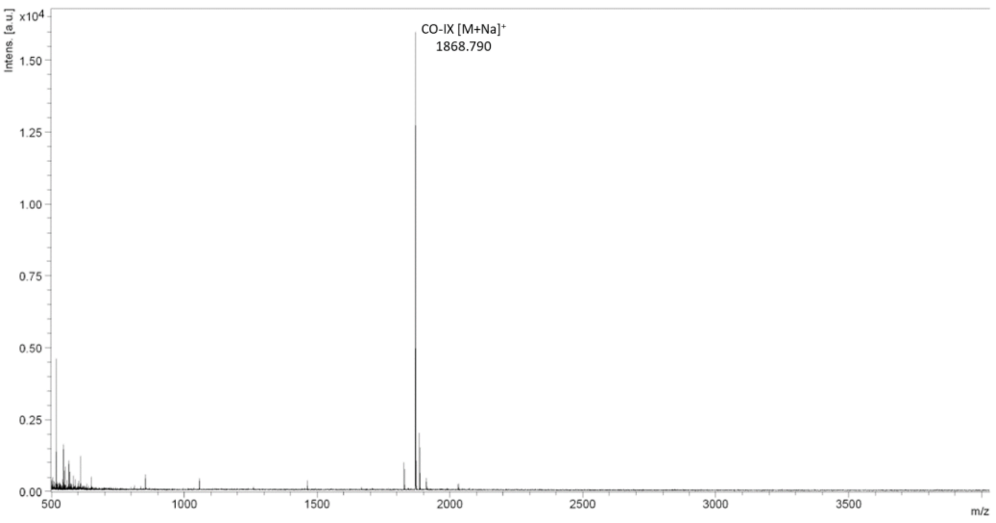

MALDI-TOF mass spectrum of purified CO-IX (positive mode, DHB matrix)

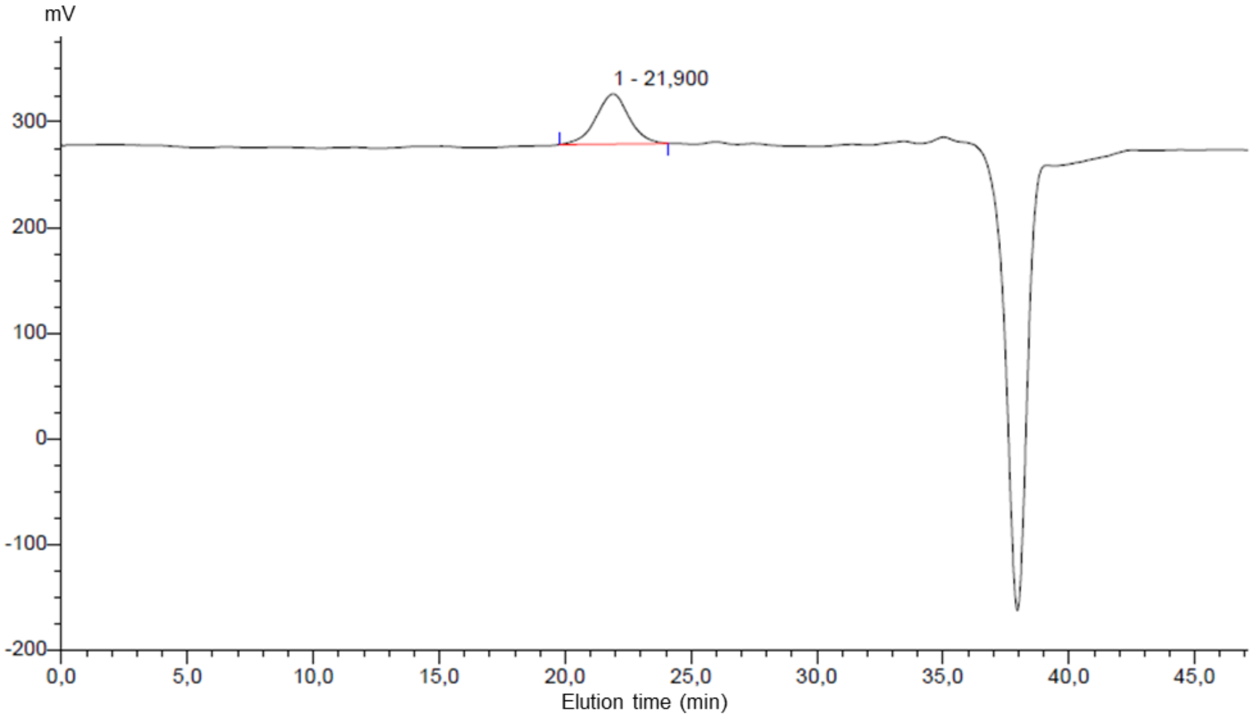

Size-exclusion chromatography profile of purified CO-IX
